# Evaluation of exposure to environmental stressors on heat‐shock protein 70 expression in normal oral keratinocyte cells

**DOI:** 10.1002/cre2.493

**Published:** 2021-09-20

**Authors:** Nafiseh Sheykhbahaei, Maryam Koopaie, Mandana Ansari

**Affiliations:** ^1^ Oral and Maxillofacial Medicine, School of Dentistry Tehran University of Medical Sciences Tehran Iran; ^2^ School of Dentistry Tehran University of Medical Sciences Tehran Iran

**Keywords:** cortisol, estrogens, heat shock proteins 70, nicotine, oral keratinocytes cell

## Abstract

**Objectives:**

This study aimed to investigate the effect of cortisol, estrogen, and nicotine on heat shock protein 70 (HSP70) expressions at the level of normal oral mucosa keratinocyte cells.

**Methods:**

In this in vitro study, keratinocytes were derived from rat oral cavity and cultured. Stressors were applied, including three groups, group 1: estrogen to simulate the postmenopausal state; group 2: cortisol to simulate psychological stress situation; group 3: nicotine to simulate smoking state. To determine the exact nature of keratinocyte cells, two surface markers, cytokeratin 18 and cytokeratin 14 were examined using the flow cytometry method. Then, the immunocytochemistry technique with three repetitions in each group was used to evaluate the HSP70 expression before and after applying the stressor.

**Results:**

HSP70 expressions in the three stressor groups (estrogen, cortisol, and nicotine) were significantly lower than in the control group (*p* = 0.0001). The HSPs expression difference between cortisol and nicotine was statistically significant (*p* = 0.0001). Based on the results of MTT analysis, the mean cell viability of oral mucosal keratinocytes in all three intervention groups decreased compared to the control group. In the cortisol and nicotine groups, cell death was significantly higher than in the control group. In the estrogen group, cell death was significantly lower than in the nicotine group (*p* > 0.05).

**Conclusions:**

The specific concentrations of cortisol, estrogen, and nicotine as stressors can effectively reduce the expression of HSP70 in normal oral mucosal keratinocytes. These phenomena can be effective in cell viability and the development of oral lichen planus.

## 1 INTRODUCTION

Oral lichen planus (OLP) is a chronic immunologic mucocutaneous inflammatory oral mucosal disease that affects 0.2%–3% of the population and the exact etiology of OLP is unknown (Alrashdan et al., 2016). Recently, induction of autoimmune reactions due to stressors has been introduced as the most important etiology in OLP (Liao et al., 2020). It is assumed that dysregulation of the genes responsible for the expression of heat shock proteins (HSPs) in the epithelial cells and its inability to suppress the immune response result in recognition of the body's own HSPs as foreign substances (Jacquemin et al., 2017). Cell exposure to physiological and environmental stressors like psychological stress, hormonal changes, smoking, hyperthermia, ischemia, anoxia, ultraviolet radiation, surgery, and mechanical pressure induce a natural immune response with a dramatic increase in the synthesis of a small group of proteins, commonly known as HSPs (Jacob et al., 2017). It seems that the role of HSPs in various diseases differs from each other. Depending on the location (intracellular or extracellular) and the type, HSPs can show different reactions to stress on the cells (Alemi et al., 2019). These proteins maintain hemostasis through different effects on the immune system and vital cell reactions (Jacob et al., 2017).

So far, the presence of HSPs 60, 70, 90, and 27 has been reported in the oral mucosa (Bayramgürler et al., 2004). There is little information about the regulatory role of HSP in oral mucosal keratinocytes under pathological and even physiological conditions. Overexpression of HSP60 increases pro‐inflammatory cytokines and induces immune system reactions (Chaiyarit et al., 1999). While overexpression of HSP70 in response to different types of stressors can play a protective role in many inflammatory and immunological diseases through reducing inflammatory cytokines and increasing cell survival (Marciniak et al., 2019; Wolf et al., 2019). The role of HSP70 in the pathogenesis of skin disorders, such as cutaneous lichen planus and psoriasis (Seifarth et al., 2018), atopic dermatitis (Ivanova et al., 2019), systemic lupus erythematosus (Jacquemin et al., 2017), and graft‐versus‐host disease has been reported (Reinhardt‐Heller et al., 2018).

Furthermore, the role of HSP60 and HSP70 in the pathogenesis of OLP (Chaiyarit et al., 1999; Sugerman et al., 1995) and oral squamous cell carcinoma (OSCC) (Neamah & Majeed, 2016) were reported in several studies. Some researchers believe that HSP expression in oral disorders such as OLP can be associated with pre‐existing inflammation in the tissue; however, others consider HSP to be the same as autoantigens in OLP (Chaiyarit et al., 1999). Dysregulation of HSP gene expression in confronting different stressors through an imbalance of cytokines secreted from involved keratinocytes and related inflammatory agents has a key role in the development, continuity, exacerbation, and characterization of OLP (Larsen et al., 2017; Pekiner et al., 2012).

Previous studies have confirmed the role of psychological stress and hormonal changes as the two major stressors in lichen planus (Agha Hosseini et al., 2016). Also, the high prevalence of OLP in women reveals the underlying hypothesis about the immunomodulatory effects of sex hormones and sex‐related genetic factors (Straub, 2007). The relationship between smoking habit and the development of autoimmune diseases has been hypothesized (Perricone et al., 2016), but the role of cigarettes in lichen planus has been less discussed so far. Regarding the controversial results of different studies about the role of HSP in the etiopathogenesis of OLP and its relation with environmental stressors, this study aimed to investigate the effect of various environmental stressors on HSP expression.

## 2 METHODS

In this in vitro study, keratinocytes were derived from rat buccal mucosa of the oral cavity and cultured. Male Wistar rats, aged 4 weeks, weighing 80–120 g, were used in this study. 50 mg kg^−1^ ketamine and 5 mg kg^−1^ diazepam were used for general anesthesia of rats. Specimen from the oral cavity was taken and stored in Dulbecco's modified Eagle medium (DMEM) culture medium with 1% penicillin–streptomycin‐fungizone for 16 h. Samples were rinsed with phosphate‐buffered saline (PBS) solution and epithelium was separated from connective tissue using trypsin at 4**°**C in 16 h. Epithelium samples were cut into 1 mm three segments and cultured in keratinocyte basal medium supplemented with Keratinocyte growth supplement and incubated at 37**°**C, 5% CO_2_, and 1%–2% humidity for at least 24 h. The cultured medium was exchanged every 2 days (culture medium purchased from sigma company [Sigma, USA]).

**Figure 1:**
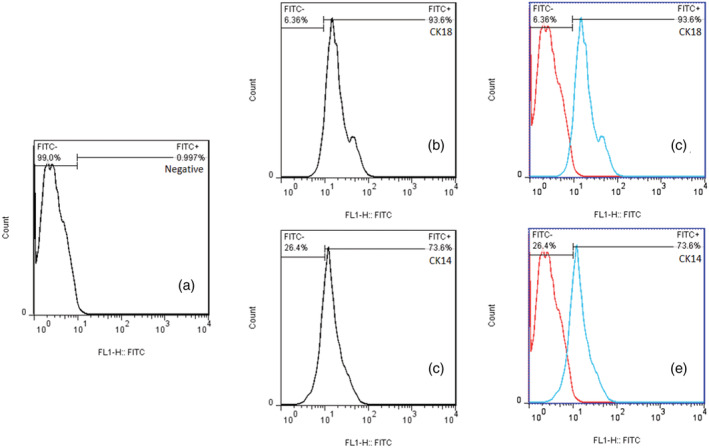
Flow cytometry of the cytokeratin pattern of oral buccal mucosa keratinocytes. The expression of cytokeratin 18 (b,c), cytokeratin 14 (d,e) and control (a) are shown

After colonies accumulation, epithelial cells trypsinized for 5 min and separated from each other. Separated keratinocytes with 105 × 1 density were cultured in a new flask. We repeated this until the second passage. Keratinocyte viability was confirmed with trypan blue exclusion. To determine the exact nature of keratinocyte cells, two surface markers, cytokeratin‐18 (CK‐18) and cytokeratin‐14 (CK‐14), were examined by flow cytometry method (Figure 1). Keratinocyte cells incubated at 37**°**C, 5% CO_2_ and keratinocyte cells were divided into three separate plates. Each plate was arranged in four wells. Stressors were applied in three wells and the fourth well was considered as the control group that was not subjected to any stressor.

Stressors applied in this study, including the following groups, group 1: estrogen to simulate the postmenopausal state (estradiol valerate 25 mg ml^−1^, code number: L04AA11); group 2: cortisol to simulate psychological stress situation (hydrocortisone 50 mg ml^−1^, injectable 2 ml vial, code number: H02AB09); group 3: nicotine to simulate smoking state (nicotine acid 100 mg, solvent tablets, code number: C10AD02).

Based on previous studies, the selected dose of each stressor was determined. In the estrogen group, two concentrations of 720 pmol l^−1^ (concentration of late follicular phase before menopause) and 140 pmol l^−1^ (concentration in menopausal phase) (Reslan & Khalil, 2012), in the cortisol group 50 mg (concentration of stress conditions) and 5 mg (concentration of non‐stress conditions) below physiological dose (Jung et al., 2014), and in the nicotine group 50 ng ml^−1^ (concentration of smoking conditions) and 4 ng ml^−1^ (concentration in non‐smoker) (Benowitz et al., 2009) were selected for comparison of cytotoxicity.

**Figure 2:**
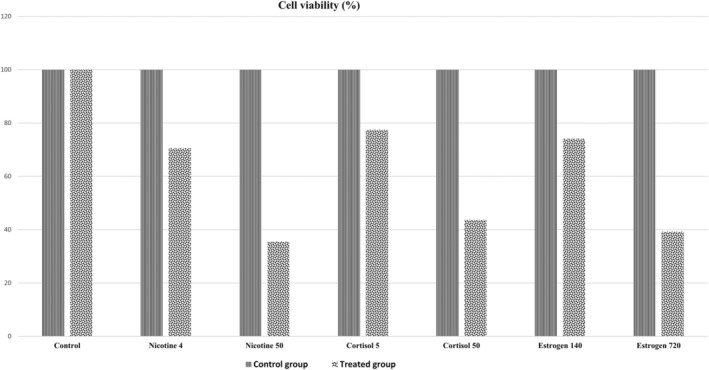
Mean cell viability in treated groups with different concentrations of stressors in comparison with the control group. MTT test was used to confirm the toxicity of selected concentrations of stressors in keratinocytes. Based on this, selected concentrations of estrogen 140 pmol l^−1^, cortisol 50 mg, and nicotine 50 ng ml^−1^ lead to less than 50% of the cells died compared to the normal doses mentioned

MTT test was used to confirm the toxicity of selected concentrations of stressors in keratinocytes. Based on this, selected concentrations of estrogen were determined to be 140 pmol l^−1^, cortisol 50 mg, and nicotine 50 ng ml^−1^. Less than 50% of the cells died compared to the normal doses mentioned (Figure 2). Keratinocyte cells stimulate with stressors in each group for 24 h. After applying the approved stressors in three plates of four wells, the immunohistochemistry (IHC) technique with three repetitions in each group was used to evaluate the HSP‐70 values before and after applying the stressor. MTT assay was used to compare the cell viability of keratinocytes in each stressor group with keratinocytes in the control group.

### 2.1 MTT; 3‐(4,5‐dimethylthylthiazol‐2‐yl)‐2,5‐diphenyltetrazolium bromide)

To perform the 3‐(4,5‐dimethylthiazol2‐yl)‐2,5‐diphenyl tetrazolium bromide (MTT) test, after 24 h, 100 ml of MTT solution was added to each well. The plates were then incubated for 3–4 h at 37**°**C. After adding this solution, the color of the environment turns blue due to the production of formazan. After this time, the plates were removed from the incubator and the top of the cells were removed. Then 1 ml of dimethyl sulfoxide (DMSO) was added to each well to dissolve the produced crystals. Then 1 ml of DMSO was added to each house to dissolve the produced crystals. After this time, all the plate wells were piped and the absorption rate was measured along the 570 nm wavelength using a spectrophotometer. The survival rate of the cells was calculated according to following Equation.

 

Percentage of survival of each sample=(absorption in treatment samples)/(absorption in control samples)×100

### 2.2 Statistical analysis

SPSS software (version 22; SPSS Inc., Chicago, IL, USA) was used to analyze the results. One‐way analysis of variance test was used to compare the mean value of HSP70 expression in normal oral keratinocyte cells between three stressor groups. All results are presented as mean ± standard deviation, and *p* ≤ 0.05 was considered statistically significant.

## 3 RESULTS

**Figure 3:**
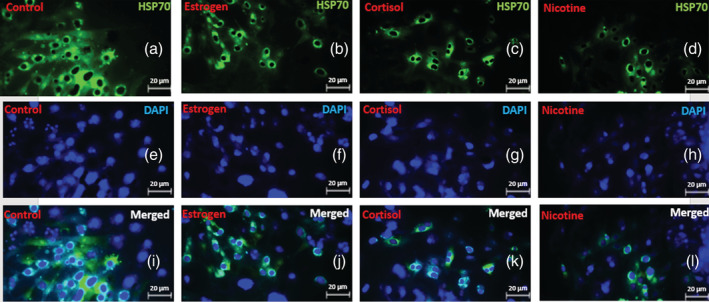
Immunohistochemically staining of oral keratinocytes in three stressor groups. Heat shock protein stained by [anti‐HSP70 antibody [5A5] (antibody 2787)] and Goat Anti‐Mouse IgG H&L (FITC) (antibody 6785) (a–d) and antinuclear antibody D9542‐1 (DAPI) (e–h) to evaluate pattern and level of expression of HSP70 protein (pattern i–l) using fluorescent microscope Olympus X400. Pattern b, c, d compared to a, j, k and l compared to i show significantly decreased staining by HSP70 antibody. Control, estrogen, cortisol and nicotine had the highest merged staining, respectively (i–l)

The present study aimed to assess the effect of estrogen 140 pmol l^−1^, cortisol [50 mg], and nicotine [50 ng ml^−1^] on HSP70 expression of normal oral keratinocyte. Immunohistochemically staining was used to assess HSP70 expression (Figure 3). The mean expression level of HSP70 in the nicotine group is 40.3, which indicates a decreased expression of this protein after keratinocyte exposure to nicotine. Mean value of HSP70 protein expression level is 56.7 in the cortisol‐stressed group and mean value e of HSP70 protein expression level in the group under estrogen stressor is 62.5 (Table 1). This reveals a decrease in protein expression after the application of estrogen stressors.

**Table 1:** The mean of heat shock protein 70 expression (%) in each group by immunocytochemistry

HSP expression	*N*	Mean value (%)	Standard deviation	Standard error	95% confidence interval (CI) for mean		Minimum	Maximum
					Lower bound	Upper bound		
Control	3	83.7667	3.33217	1.92383	75.4891	92.0442	80.20	86.80
Cortisol 50	3	56.7000	1.93132	1.11505	51.9023	61.4977	54.60	58.40
Estrogen 140	3	62.5000	2.90517	1.67730	55.2832	69.7168	59.70	65.50
Nicotine 50	3	40.3000	2.35797	1.36137	34.4425	46.1575	38.30	42.90

The data were expressed as mean ± standard error of the mean and analyzed by one‐way analysis of variance and Tukey's post hoc test.

**Figure 4:**
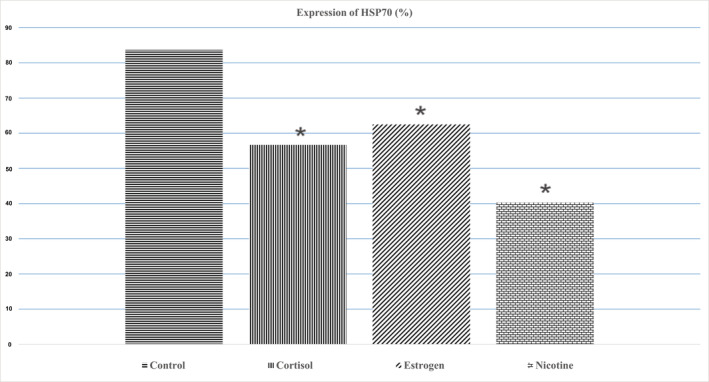
The mean expression (%) of HSP70 in treated groups in comparison with control group. Following culture of the keratinocyte cells in the presence of different stressors, immunohistochemically staining revealed a significant reduction in expression of HSP70 compared to the untreated control. Each value represents the mean for three independent experiments. Data analyzed by one‐way analysis of variance and Tukey's post hoc test. * indicate a significant difference with the control (*p* < 0.05)

Using a one‐way analysis of variance test for comparison of the mean value of HSP70 on normal oral keratinocytes revealed that HSP70 expression in three stressor groups (estrogen, cortisol, and nicotine) was significantly lower than the control group (*p* = 0.000). The HSP70 expression differences between group 1 (estrogen) and group 2 (cortisol) are not statistically significant (*p* = 0.110). The HSP70 expression difference between group 1 (estrogen) and group 3 (nicotine) is statistically significant (*p* = 0.000). The HSP70 expression difference between group 2 (cortisol) and group 3 (nicotine) is statistically significant (*p* = 0.0001) (Figure 4). Based on the results of MTT analysis, the mean cell viability of oral mucosal keratinocytes in all three intervention groups decreased compared to the control group. In the cortisol and nicotine groups, cell death was significantly higher than in the control group. In the estrogen group, cell death was significantly lower than in the nicotine group (*p* > 0.05) (Table 2).

**Table 2:** The mean difference of cell viability in stressor groups. In the cortisol and nicotine groups, cell viability was significantly lower than to the control group. In the estrogen group, cell viability was significantly higher than in the nicotine group

MTT groups		Mean difference	Standard error	*p* value	95% confidence interval (CI)	
					Lower bound	Upper bound
Estrogen 140	Cortisol 50	31.38483	9.85113	0.075	−2.2527	65.0224
	Nicotine 50	39.59638^a^	9.85113	0.017	5.9589	73.2339
	Control	−24.91301	9.85113	0.221	−58.5505	8.7245
Cortisol 50	Nicotine 50	8.21155	9.85113	0.977	−25.4260	41.8491
	Estrogen 140	−31.38483	9.85113	0.075	−65.0224	2.2527
	Control	−56.29784^a^	9.85113	0.001	−89.9354	−22.6603
Nicotine 50	Estrogen 140	−39.59638^a^	9.85113	0.017	−73.2339	−5.9589
	Cortisol 50	−8.21155	9.85113	0.977	−41.8491	25.4260
	Control	−64.50939^a^	9.85113	0.000	−98.1469	−30.8719

The data were expressed as mean ± standard error of the mean and analyzed by one‐way analysis of variance and Tukey's post hoc test.

^a^ Indicates a significant difference with other groups at *p* < 0.05.

## 4 DISCUSSION

To the best of our knowledge, there is no study to examine changes in HSP expression under environmental stressors in normal oral mucosal keratinocytes as a predisposing factor in OLP. According to the present study, environmental stressors including certain concentrations of cortisol, nicotine, and estrogen have led to a significant reduction in HSP70 expression at the level of normal oral mucosal keratinocytes and a decrease in cell viability. An interesting finding of this study is the effect of nicotine on HSP70 expression and its possible role in the pathogenesis of lichen planus. To the best of our knowledge, up to now, smoking has not been identified as the main risk factor for OLP. However, the role of tobacco and its products, especially nicotine in the etiopathogenesis of oral cancer and other autoimmune diseases, is well established (Perricone et al., 2016). Besides decreased HSP70, upregulation of inflammatory cytokines, neoangiogenesis, vascular endothelial growth factor (VEGF) secretion, and the production of oxygen‐free radicals is from the nicotine‐dependent mechanisms that could be involved in the pathogenesis of OLP. In addition, our findings confirm the role of stress and estrogen in OLP etiopathogenesis.

The role of psychological stress and excessive cortisol secretion in oral disease such as OLP (Gaur et al., 2018) has been reported in several studies. Low levels of estrogen in post‐menopausal women lead to induction of the cellular immune system (Gameiro et al., 2010), increased activity of T‐Cytotoxic cells increases the production of inflammatory cytokines by activation of nuclear factor kappa B (NF‐κB) (Zhang et al., 2020), induce oxidative stress (Bourgonje et al., 2020), and decrease the production of anti‐inflammatory cytokines (Humberto et al., 2018; Upadhyay et al., 2010). The immunomodulatory, anti‐inflammatory, induction of cell proliferation, and cell protection effects of HSP70 have been reported in some studies. Two studies in 2019, shown inhibition of HSP70 in periodontal ligament (PDL) cells caused decreased cell proliferation, wound healing, colony formation, and a significant increase in inflammatory cytokines such as cell adhesion molecules, cell apoptosis, and necrosis and osteoclastic differentiation. These data confirmed the cell‐protective and anti‐inflammatory effects of HSP70 (Marciniak et al., 2019; Wolf et al., 2019). Increased expression of intercellular adhesion molecules (ICAM) (Malarkodi & Sathasivasubramanian, 2015) and other adhesion molecules are from non‐specific effective mechanisms in the OLP pathogenesis. Adhesion molecules such as E‐selection and ICAM‐1 are effective in increasing leukocyte migration and infiltration in and around the basement membrane. Some researchers suggested that upregulation of HSP70 in respiratory mucosa in exposure to pepsin revealed the protective effect of HSP70 (Wang et al., 2019).

Also, a decrease in HSP70 expression was reported in primary fibroblast cells cultured from idiopathic pulmonary fibrosis compared with normal lung tissue cells. Decreased HSP70 through increased extracellular matrix metalloproteinase (MMP) is effective in pulmonary fibrosis (Sellares et al., 2019). Increase production of MMP and MMP‐like collagenases (Sugerman et al., 2002) is involved in activation, migration, proliferation, and differentiation of T cells and their infiltration into the superficial lamina propria, destruction of the basement membrane, infiltration of immune cells into the epidermis, upregulation of tumor necrosis factor‐alpha (TNF‐α) and keratinocyte apoptosis are from the main process that has a critical role in OLP (Tsai et al., 2009).

Our finding reveals that decreased HSP70 expression in oral keratinocytes can lead to necrosis and cell death, which are one of the main microscopic features of OLP (Ramakrishnan et al., 2020). Band‐like lymphocytic infiltration adjacent to the basement membrane and subsequent hydropic degeneration of basal epithelial cells and degeneration of the basement membrane occurs in OLP (Escudier et al., 2007). Also, induction of apoptosis in epidermal cells, following the suppression of HSP70 has been reported (Matsuda et al., 2010). It seems that downregulation of HSP70 through induction of inflammatory processes and cell death can be effective in OLP. This finding is confirmed by data from previous studies. The lower levels of expression of HSP70 in the epithelium of cutaneous lichen planus, especially the basal layer compared to normal skin (Bayramgürler et al., 2004) and OLP tissue in comparison with healthy mucosa showed the effective role of HSP70 in OLP. Also, Albanidou‐Farmaki et al. showed a significant increase in HSP70 in reticular lichen planus compared to the control group. However, this finding was not seen in erosive OLP. This may indicate the immunomodulatory role of this protein in the chronicity of OLP. However, some other studies have reported an increase in HSP70 expression in OLP samples compared to healthy mucosa (Sugerman et al., 1995). A study by Chaiyarit et al. did not show any significant difference in HSP70 expression between OLP and fibroma tissues (Chaiyarit et al., 1999). This discrepancy in results may be related to differences in fixation, retrieval antigen, and primary antibodies used in the IHC technique (Chaiyarit et al., 1999).

It is noteworthy that downregulation of HSP70, because of cell necrosis induction, is probably a control phenomenon to reduce the risk of malignant transformation in OLP. However, if the tissue goes to cancer and dysplasia, the process is probably reversed. An increase in HSP70 leads to the loss of cell cycle control, reduction of cell apoptosis, and cancer induction (Neamah & Majeed, 2016). HSP70 expression in normal mucosa was reported to be approximately 20%, in inflammatory‐autoimmune lesions 10%–20%, in dysplasia (81%), and OSCC (85%) (Neamah & Majeed, 2016).

Based on the available evidence, it seems that HSP70 has a proliferative effect that contributes to the remodeling and regeneration of normal oral mucosal cells (Wolf et al., 2019). In general, the balance of different types of HSP in normal conditions maintains oral health, and imbalances in their ratios due to different stressors can lead to pathological conditions and various diseases. We recommend further tissue studies, especially assessment of the cytokines, biomarkers, and other HSPs associated with OLP pathogenesis to reveal the relationship between stressors and OLP pathogenesis.

## 5 CONCLUSION

The specific concentrations of cortisol, estrogen and nicotine as stressors can be effective in reducing the expression of HSP70 in normal oral mucosal keratinocytes. These phenomena can be effective in cell viability and the development of OLP.

**Abbreviations**
CK‐14: cytokeratin‐14
CK‐18: cytokeratin‐18
DMEM: Dulbecco's modified Eagle medium
DMSO: dimethyl sulphoxide
HSP 70: heat shock protein 70
HSP: heat shock protein
ICAM: intercellular adhesion molecules
IHC: immunocytochemistry
MMP: matrix metallo proteinase
NF‐κB: nuclear factor kappa B
OLP: oral lichen planus
OSCC: oral squamous cell carcinoma
PBS: phosphate buffered saline
PDL cell: periodontal ligament cell
TNF‐α: tumor necrosis factor‐alpha
VEGF: vascular endothelial growth factor

## CONFLICT OF INTEREST

The authors declare no conflict of interest.

## AUTHOR CONTRIBUTIONS

Nafiseh Sheykhbahaei and Maryam Koopaie planned the study, designed the experiments and wrote the manuscript. Mandana Ansari performed the experiments and analyzed the data. All authors read and approved the final manuscript.

## ETHICS STATEMENT

This study does not involve human participants and animals, human and animal data, or human and animal tissues and informed consent was therefore not required. This study was approved by Tehran University of Medical Sciences Ethical Committee (Ethics code: IR.TUMS.DENTISTRY.REC.1397.157).

## Data Availability

The data that support the findings of this study are available from the corresponding author upon reasonable request.
